# Are pumas subordinate carnivores, and does it matter?

**DOI:** 10.7717/peerj.4293

**Published:** 2018-01-24

**Authors:** L. Mark Elbroch, Anna Kusler

**Affiliations:** 1 Panthera, New York, NY, United States of America; 2 Department of Biology, Pace University Pleasantville/Briarcliff, Pleasantville, NY, United States of America

**Keywords:** Puma concolor, Subordinate, Dominance, Competition, Gray wolf, Grizzly bear, American black bear, Jaguar, Apex predators

## Abstract

**Background:**

Interspecific competition affects species fitness, community assemblages and structure, and the geographic distributions of species. Established dominance hierarchies among species mitigate the need for fighting and contribute to the realized niche for subordinate species. This is especially important for apex predators, many of which simultaneous contend with the costs of competition with more dominant species and the costs associated with human hunting and lethal management.

**Methods:**

Pumas are a widespread solitary felid heavily regulated through hunting to reduce conflicts with livestock and people. Across their range, pumas overlap with six apex predators (gray wolf, grizzly bear, American black bear, jaguar, coyote, maned wolf), two of which (gray wolf, grizzly bear) are currently expanding in North America following recovery efforts. We conducted a literature search to assess whether pumas were subordinate or dominant with sympatric apex predators, as well as with three felid mesocarnivores with similar ecology (ocelot, bobcat, Canada lynx). We also conducted an analysis of the spatial distributions of pumas and their dominant sympatric competitors to estimate in what part of their range, pumas are dominant versus subordinate.

**Results:**

We used 64 sources to assess dominance among pumas and other apex predators, and 13 sources to assess their relationships with felid mesocarnivores. Evidence suggested that wolves, grizzly bears, black bears, and jaguars are dominant over pumas, but that pumas are dominant over coyotes and maned wolves. Evidence suggested that pumas are also dominant over all three felid mesocarnivores with which they share range. More broadly, pumas are subordinate to at least one other apex carnivore in 10,799,252 (47.5%) of their 22,735,268 km^2^ range across North and South America.

**Discussion:**

Subordinate pumas change their habitat use, suffer displacement at food sources, likely experience increased energetic demands from harassment, exhibit increased starvation, and are sometimes directly killed in competitive interactions with dominant competitors. Nevertheless, we lack research clearly linking the costs of competition to puma fitness. Further, we lack research that assesses the influence of human effects simultaneous with the negative effects of competition with other sympatric carnivores. Until the time that we understand whether competitive effects are additive with human management, or even potentially synergistic, we encourage caution among managers responsible for determining harvest limits for pumas and other subordinate, apex carnivores in areas where they are sympatric with dominant species. This may be especially important information for managers working in regions where wolves and brown bears are recolonizing and recovering, and historic competition scenarios among multiple apex predators are being realized.

## Introduction

The outcome of interspecific competition affects species fitness, community assemblages and structure, and the geographic distributions of species (Schoener, 1983; Polis & Holt, 1992; Le Bourlot, Tully & Claessen, 2014; Dröge et al., 2017). Much of competition is indirect, and competitors generally assess the competitive abilities (e.g., resource holding potential; Parker & Stuart, 1976) of their opponents before deciding to engage them, resulting in established species hierarchies that mitigate the need for fighting (Arnott & Elwood, 2008). In fact, the established “superiority” (Crombie, 1947) of one species over another contributes to Hutchinson’s (1957) differentiation between the fundamental and realized niche. A species’ enemies include both predators and competitors, both of which limit an animal’s distribution to its realized niche (e.g., wild dogs, *Lycaon pictus*, in Dröge et al., 2017). This is especially important for carnivores in multi-carnivore guilds, in which competition contributes to intraguild killing (Polis & Holt, 1992; Donadio & Buskirk, 2006) and whether a species self-regulates its population through various social mechanisms or is instead regulated by another predator (Wallach et al., 2015).

Carnivores disproportionately influence ecological functions in natural systems and are culturally important (Ripple et al., 2014). Most carnivores, however, are also feared and persecuted because of perceived and real threats to human safety and because they kill livestock, household pets, and game species we value (Ordiz, Bischof & Swenson, 2013; Ripple et al., 2014; Elbroch, Feltner & Quigley, 2017). Not all carnivores are ecologically equivalent, however, and size betrays stark differences in small versus large carnivores. Carbone, Teacher & Rowcliffe (2007) found that carnivores <20 kg exhibited different hunting strategies and energetic budgets than carnivores >20 kg, and Wallach et al. (2015) found that carnivores <15 kg exhibited additional differences in life history strategies than those species >15 kg. Specifically, Wallach et al. (2015) reported that carnivores >15 kg self-regulate through social mechanisms and density-dependence, whereas smaller carnivores are generally limited by predation by larger carnivores. Wallach et al. (2015) subsequently used 15 kg to differentiate between “apex predators” and smaller carnivores. For our review, we also utilized the 15-kg cutoff.

Apex predators, too, are not ecologically equivalent, and vary in size, morphology, weapons, and life history strategies (Kleiman & Eisenberg, 1973; Bekoff, Daniels & Gittleman, 1984; Sandell, 1989). These differences have resulted in established, though sometimes variable, dominance hierarchies, as well as the unique morphology and behaviors utilized by subordinate species coping with the pressures of interspecific competition. For example, pumas (*Puma concolor*) and cheetahs (*Acinonyx jubatus*) appear to withstand high levels of kleptoparatism without obvious influences on their fitness (Scantlebury et al., 2014; Elbroch et al., 2017a), and coyotes (*Canis latrans*), wild dogs (*Lycaon pictus*), leopards (*Panthera pardus*), and cheetahs utilize temporal and spatial competition refuges and other movement strategies to mitigate interactions with dominant competitors (Durant, 1998; Arjo & Pletscher, 1999; Vanak et al., 2013; Dröge et al., 2017).

The position a species occupies in a dominance hierarchy of a multi-carnivore guild influences its ecology (e.g., foraging strategies and temporal space use; Dröge et al., 2017), but external ecological factors, such as resource distributions, also influence the strategies subordinate species utilize to coexist with dominant competitors, or whether they exhibit subordinate behaviors at all (Karanth et al., 2017). Evidence strongly supports the theory that size determines the outcomes of competitive interactions and the structure of dominance hierarchies; in general, larger carnivores dominate smaller carnivores (Schoener, 1983; Donadio & Buskirk, 2006; De Oliveira & Pereira, 2014). Nevertheless, this is not always the case. Striped skunks (*Mephitis mephitis*), bobcats (*Lynx rufus*), and wolverines (*Gulo gulo*) compensate for their smaller size by being more aggressive and wielding unique weapons (Krebs et al., 2004; Allen et al., 2016).

Determining whether or not a species is dominant or subordinate may also be important in applied conservation and management. Most of the world’s apex predators are hunted by humans, often for trophies (Ordiz, Bischof & Swenson, 2013), and whereas carnivores have evolved strategies to cope with interspecific competition, they are less prepared to withstand the intensity of modern human hunting (Cooley et al., 2009; Darimont et al., 2015). Humans differ from other predators in that they disproportionately target other carnivores and adult animals, which constitute the reproductive capital of populations (Darimont et al., 2015). In the case of subordinate species already under pressures of interspecific competition, human impacts may lead to unexpected population fluctuations or declines. For example, following the recovery of a tiger population in India, subordinate leopards shifted their diet, increased their conflicts with people through livestock depredation, and subsequently suffered rapid declines in their numbers (Harihar, Pandav & Goyal, 2011).

Pumas are a widespread, solitary felid heavily regulated through sport hunting to mitigate conflicts with livestock and people (Treves, 2009; Murphy & MacDonald, 2010; Mattson, 2014). Across their range in North and South America, pumas overlap with six other apex predators (as defined by Wallach et al., 2015). Grizzly (or “brown”) bear (*Ursus arctos*) and gray wolf (*Canis lupus*) populations are currently expanding in North America and increasingly overlapping managed puma populations already under pressure from human harvest. If pumas are dominant over bears and wolves, the expansion of these potential competitors should not influence puma fitness or population dynamics significantly. But, if pumas are subordinate to one or both of these recovering species, conservation mangers may need to adjust human harvest to accommodate for the additional ecological stress of new competition. The first step, then, is to determine whether pumas are indeed a subordinate carnivore, and if so, ascertain to which apex carnivores they are subordinate.

In this paper, we had three objectives: First, we reviewed the published literature to gather empirical evidence for whether pumas are dominant, subordinate, or equal in their competitive interactions with the following apex predators: grizzly bear, American black bear (*Ursus americanus*), gray wolf, coyote, maned wolf (*Chrysocyon brachyurus*), and jaguar (*Panthera unca*). Following established theory, we predicted that size would determine competitive outcomes, and that social carnivores and larger carnivores would dominate pumas (Donadio & Buskirk, 2006; De Oliveira & Pereira, 2014). Second, we gathered information on interactions with three ecologically-similar felid mesocarnivores (bobcat, Canada lynx, *Lynx canadensi*s, and ocelot, *Leopardus pardalis*) with which the potential for competition with pumas over resources is also likely. Third, we assessed the extant spatial distribution of pumas, and quantified in what portion of their range they are the dominant predator versus a subordinate under other sympatric apex predators. Managers and scientists can then refer to this map as they consider hunting limits, conservation priorities, and research questions for the species.

## Survey Methodology

### Literature search

We searched for evidence of dominance and subordination among species interactions in the published literature. We conducted independent searches using Google Scholar and Web of Science, utilizing puma-species pairings and the following keywords: puma, *puma concolor*, cougar, mountain lion, jaguar, *Panthera onca*, bear, black bear, grizzly bear, *Ursus*, wolf, wolves, gray wolf, *Canis lupus*, coyote, *C. latrans*, ocelot, *Leopardus pardalis*, bobcat, *Lynx rufus*, maned wolf, *Chrysocyon brachyurus*, Canada lynx, *L. canadensis*. We are confident this approach captured most publications in English, but we are less sure what percent of Spanish publications were identified successfully.

### Types of competition and awarding dominance

We sought evidence for outcomes from the following four types of interspecific interactions:

(1) Exploitation competition (EC) describes indirect interactions in which a dominant species superior at gathering, using, or acquiring a shared resource, reduces the availability of the shared resource for the subordinate species (Tilman, 1982). For example, if a subordinate species shifts its diet in the presence of a dominant, the change may be interpreted as exploitation competition (Hayward & Kerley, 2008).
(2) Interference competition (IC), defined as aggression or direct contests in which a dominant species blocks a subordinate species from a resource (Case & Gilpin, 1974). In natural systems, it can be difficult to interpret between exploitive and interference competition (Schoener, 1983). For example, pumas reduce their intake of elk (*Cervus canadensis*) in the presence of wolves (e.g., Kortello, Hurd & Murray, 2007), but it is unclear whether wolves are better at killing this prey or whether wolves are directly (chasing pumas) or indirectly blocking pumas from elk, by forcing elk to shift to habitats in which pumas hunt less often. IC may also elevate to physical contests in which the subordinate species is killed, described next.
(3) Interspecific competitive killing (CK), defined as a dominant species killing a subordinate species, but not eating them (Lourenço et al., 2013).
(4) Intraguild predation (IGP), defined as a dominant predator killing and consuming a subordinate predator (Holt & Polis, 1997; Lourenço et al., 2013).

Differentiating between these four categories, however, proved challenging. Schoener (1983) emphasized the difficulty in interpreting mechanisms of competition (EC vs. IC) from field data and acknowledged that both mechanisms may contribute to specific animal interactions and behaviors. Likewise, differentiating CK from IGP required knowledge on whether the prey (subordinate carnivore) was consumed in its entirety (Lourenço et al., 2013), and this information was lacking in most published papers we read. Therefore, we simplified the four categories to two: (1) non-lethal competition (NLC), inclusive of all forms of EC and IC; and (2) lethal competition (LC), inclusive of CK and IGP.

We tallied the number of publications that provided evidence for whether pumas were dominant or subordinate in interactions with each sympatric carnivore species, and awarded dominance to the species in the pairing with the most evidence (highest number of publications) supporting dominance.

### Geographic analysis to determine where pumas are dominant versus subordinate

Based upon our results determining which species were dominant over pumas and which were subordinate, we assessed coarse spatial patterns across the extant range of pumas. We acquired distribution maps for pumas and the species to which they were subordinate from the International Union for Conservation of Nature (IUCN) (Wildlife Conservation Society, 2008; IUCN, 2010; IUCN, 2015; IUCN, 2016; IUCN SSC Bear Specialist Group, IUCN and IBA, 2017). IUCN range maps are polygons for Geographic Information Systems (GIS) that represent extant ranges where a species is likely to occur, based upon known occurrences of the species, habitat preferences and subsequent modeling, and other expert knowledge of the species and its range (IUCN, 2017). We utilized the Union tool in ArcGIS 10.0 to create a single distribution for the dominant species, removing any redundancy where species overlapped. Then we used the Overlay tool to quantify in what proportion of a their range pumas are dominant versus subordinate to one or more competitors.

## Results

**Figure 1:**
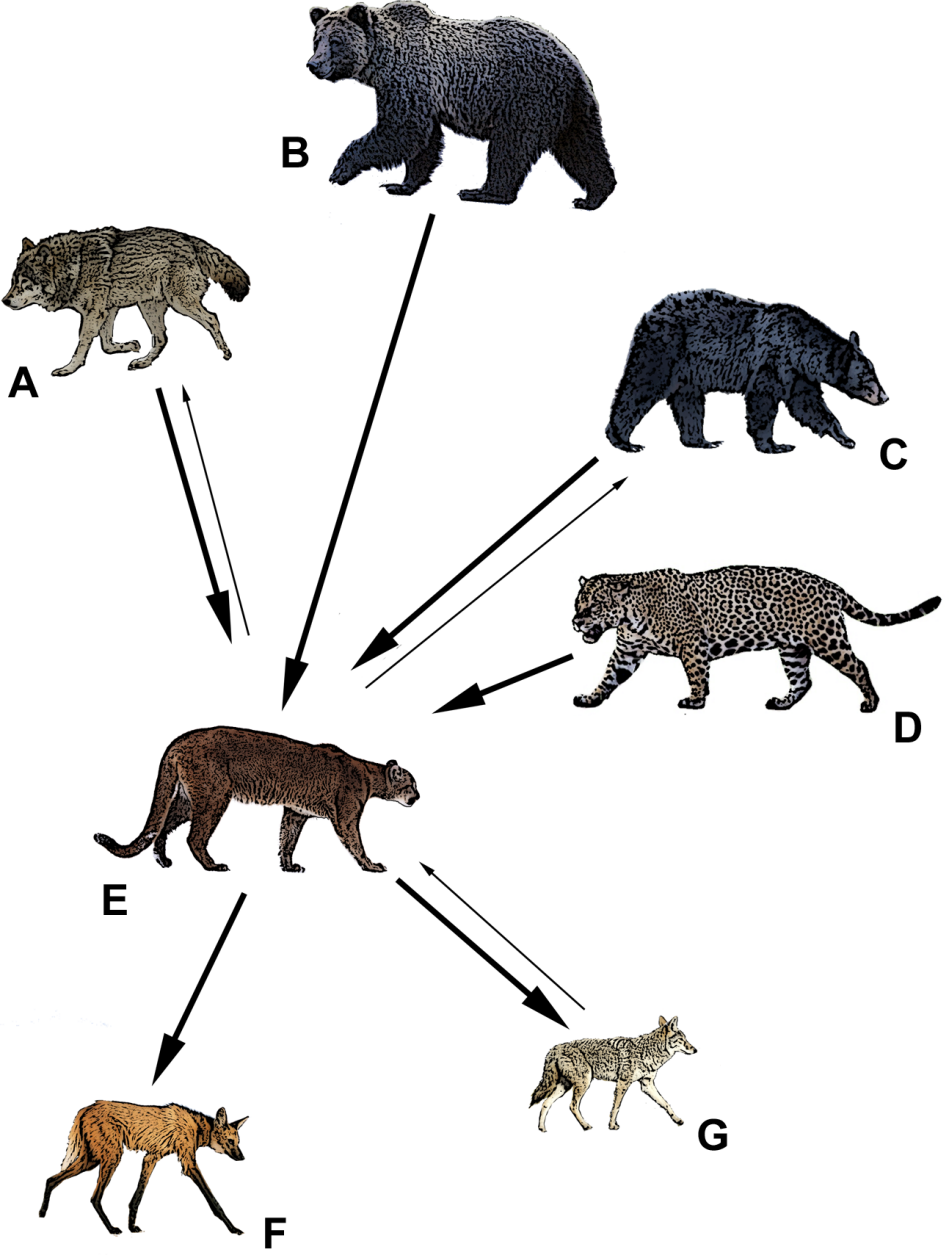
The apex predators of North and South America and their relative competitive relationship with pumas (E). Bold arrows denote dominance, and point from the dominant species to the subordinate. Thin arrows denote some evidence to the contrary. (A) gray wolf (*Canis lupus*), (B) grizzly bear (*Ursus arctos*), (C) American black bear (*Ursus americanus*), (D) jaguar (*Panthera unca*), (E) puma (*Puma concolor*), (F) maned wolf (*Chrysocyon brachyurus*), (G) coyote (*Canis latrans*). Drawings by Mark Elbroch.

Our initial searches in Web of Science and Google Scholar yielded 72 potential papers that in turn yielded another 15 scientific accounts (e.g., old graduate theses and state reports) for our review of competition between pumas and sympatric apex predators. In the end, we utilized a refined list of 64 distinct sources in our review; those that were discarded did not address competition between apex predators or were redundant publications of earlier findings. We found evidence that wolves dominated pumas in 78% (*n* = 18) of 23 sources, grizzly bears dominated pumas in 100% of 4 sources, black bears dominated pumas in 67% (*n* = 6) of 9 sources, jaguars dominated pumas in 60% (*n* = 15) of 25 sources, coyotes dominated pumas in 19% (*n* = 3) of 16 sources, and zero examples in which maned wolves dominated pumas (Table 1; Fig. 1). In contrast, we found evidence that pumas dominated wolves in 22% (*n* = 5) of 23 sources, black bears in 44% (*n* = 4) of 9 sources, coyotes in 81% (*n* = 13) of 16 sources, and maned wolves in 67% (*n* = 2) of 3 sources (Table 2; Fig. 1). We also found 10 sources that did not detect dominance in either species where jaguars and pumas were sympatric (Table 2).

**Table 1:** Puma subordination. Evidence of puma subordination to sympatric apex carnivores, the number of sources (*n*) supporting the conjecture, and the citations of the sources.

Species pairing	*n*	NLC	LC
Wolf dominant over puma	18	Puma spatial displacement: Riley, Nesslage & Maurer (2004), Kortello, Hurd & Murray (2007), Bartnick et al. (2013), Lendrum et al. (2014) Puma displacement at carcasses and kleptoparasitism of puma kills: USFW (1995), Ruth & Hornocker (1996), Kunkel et al. (1999), Hornocker Wildlife Institute (2001), Akenson, Akenson & Quigley (2005), Kortello, Hurd & Murray (2007), Ruth & Buotte (2007), Elbroch et al. (2017c), Puma prey switching due to wolves: Kortello, Hurd & Murray (2007), Bartnick et al. (2013), Elbroch et al. (2015)	Direct killing of pumas: Schmidt & Gunson (1985), White & Boyd (1989), Boyd & Neal (1992), Kunkel et al. (1999), Jimenez et al. (2006), Kortello, Hurd & Murray (2007), Ruth (2004a), Ruth & Buotte (2007), Elbroch et al. (2015) Puma starvation attributed to competition: Ruth (2004a), Kortello, Hurd & Murray (2007) and Elbroch et al. (2015)
Grizzly bear dominant over puma	4	Puma displacement at carcasses and kleptoparasitism of puma kills: Murphy et al. (1998), Ruth & Buotte (2007); LM Elbroch & HB Quigley, 2018, unpublished data	Direct killing of pumas: Ruth (2004b)
Black bear dominant over puma	6	Puma displacement at carcasses and kleptoparasitism of puma kills Murphy et al. (1998), Ruth (2004b), Allen et al. (2014a), Elbroch et al. (2014) and Allen et al. (2015)	Direct killing of pumas: LM Elbroch & HB Quigley, 2018, unpublished data (black bear killed and consumed three kittens in same litter)
Jaguar dominant over puma	15	Spatial and temporal displacement of pumas: Schaller & Crawshaw Jr (1980), Emmons (1987), Scognamillo et al. (2003), Moreno, Kays & Samudio Jr (2006)De Azevedo (2008), Harmsen et al. (2009), Paviolo et al. (2009), Romero-Munoz et al. (2010), Sollmann et al. (2012), Palomares et al. (2016), Palomares et al. (2017) Change in puma diet when sympatric with jaguars: Moreno, Kays & Samudio Jr (2006)	Direct killing of pumas: Crawsha Jr & Quigley (1984), Crawshaw & Quigley (1991), Harmsen et al. (2009), Ruth & Murphy (2010), De Oliveira & Pereira (2014)
Coyote dominant over puma	3	Puma displacement at carcasses and kleptoparasitism of puma kills: Harrison (1990), Stanford (2013)	Direct killing of pumas: Logan & Sweanor (2001)

**Notes.**

NLC non-lethal competition LC lethal competition

**Table 2:** Puma dominance and equality. Evidence of (1) puma dominance over and (2) equality to sympatric apex carnivores, the number of sources (*n*) supporting the conjecture, and the citations of the sources.

Species pairing	*n*	NLC	LC
**Dominance**			
Puma dominant over wolf	5	–	Direct killing of wolves: Schmidt & Gunson (1985), Ruth (2004a), Mexican Wolf Blue Range Adaptive Management Oversight Committee and Interagency Field Team (2005), Jimenez et al. (2008); LM Elbroch & HB Quigley, 2018, unpublished data (1 subadult wolf killed and consumed by an adult female puma)
Puma dominant over grizzly bear	0	–	–
Puma dominant over black bear	4	–	Direct killing of black bears: Cunningham, Gustavson & Ballard (1999), Elbroch et al. (2014), Nichols (2017); LM Elbroch & HB Quigley, 2018, unpublished data (adult female puma kills and consumes black bear cub of the year)
Puma dominant over jaguar	0	**-**	–
Puma dominant over coyote	13	Spatial and temporal displacement of coyotes: Koehler & Hornocker (1991), Wang, Allen & Wilmers (2015), Mahoney (2017)	Direct killing of coyotes Ackerman, Lindzey & Hemker (1984), Boyd & O’Gara (1985), Arjo (1998), Murphy et al. (1998), Koehler & Hornocker (1991), Atwood (2006), Ruth & Buotte (2007), De la Torre & De la Riva (2009), Knopff et al. (2010), Lowrey, Elbroch & Broberg (2016); LM Elbroch & HB Quigley, 2018, unpublished data (multiple pumas killing and consuming multiple coyotes)
Puma dominant over maned wolf	2	–	Direct killing of maned wolves: Mazzolli (2009), De Oliveira & Pereira (2014)
**Equality**			
Jaguar and puma equal	10	Inferred because spatial/temporal activity patterns were similar: Taber et al. (1997), Scognamillo et al. (2003), Foster, Harmsen & Doncaster (2010), Davis, Kelly & Stauffer (2011), Foster et al. (2013), Hernández-SaintMartín (2013), Gutiérrez-González & López-González (2017) Inferred because diet patterns were dissimilar: Scognamillo et al. (2003), Novack et al. (2005), De Azevedo (2008), Hernández-SaintMartín (2015)	–
Maned wolf and puma equal	1	Inferred because spatial/temporal activity patterns were similar: Trolle et al. (2007)	–

**Notes.**

NLC non-lethal competition LC lethal competition

We utilized 13 distinct sources to assess puma interactions with three large sympatric felid mesocarnivores and the literature suggested that pumas were dominant over all three species. We found three sources reporting pumas killing ocelots (Crawshaw, 1995; López-González & González-Romero, 1998; Martins, Quadros & Mazzolli, 2008), but two additional references that indicated pumas and ocelots may not influence each other (Davis, 2009; Massara et al., 2016). We only found one reference to pumas killing Canada lynx (Ruth & Murphy, 2010), and nothing further about puma-lynx competition. We found six papers documenting puma predation of bobcats (Ackerman, Lindzey & Hemker, 1984; Burton, Perez & Tovar, 2003; Hass, 2009; Koehler & Hornocker, 1991; Lopez-González, 1994; Lowrey, Elbroch & Broberg, 2016), and three references reporting spatial displacement of bobcats by pumas (Koehler & Hornocker, 1991; Burton, Perez & Tovar, 2003; Lewis et al., 2015).

We were unable to analyze whether weight ratios correlated with dominance because of complications due to extreme sexual dimorphism among pumas and other carnivores (and sources not always reporting the sex of the animals involved in interactions), and the fact that social carnivores should likely be analyzed as the combined weight of all members in an interaction (e.g., wolf pack); group size in interactions with social species was often lacking as well. Overall, the general pattern was suggestive that the size difference between pumas and other apex predators does correlate with dominance. For example, evidence that jaguars are dominant is strongest in areas where jaguars are large and weigh considerably more than pumas, but more ambiguous where the two species are more similar in size (López-González & González-Romero, 1998; Gutiérrez-González & López-González, 2017). Wolf packs also appear to have a clear advantage over individual pumas, but one-on-one, the outcome of competitive interactions between a wolf and a puma is less certain (Ruth & Murphy, 2010) or dependent upon differences in age (e.g., wolves killing puma kittens; Elbroch et al., 2015).

### Geographic analysis to determine where pumas are dominant versus subordinate

**Figure 2:**
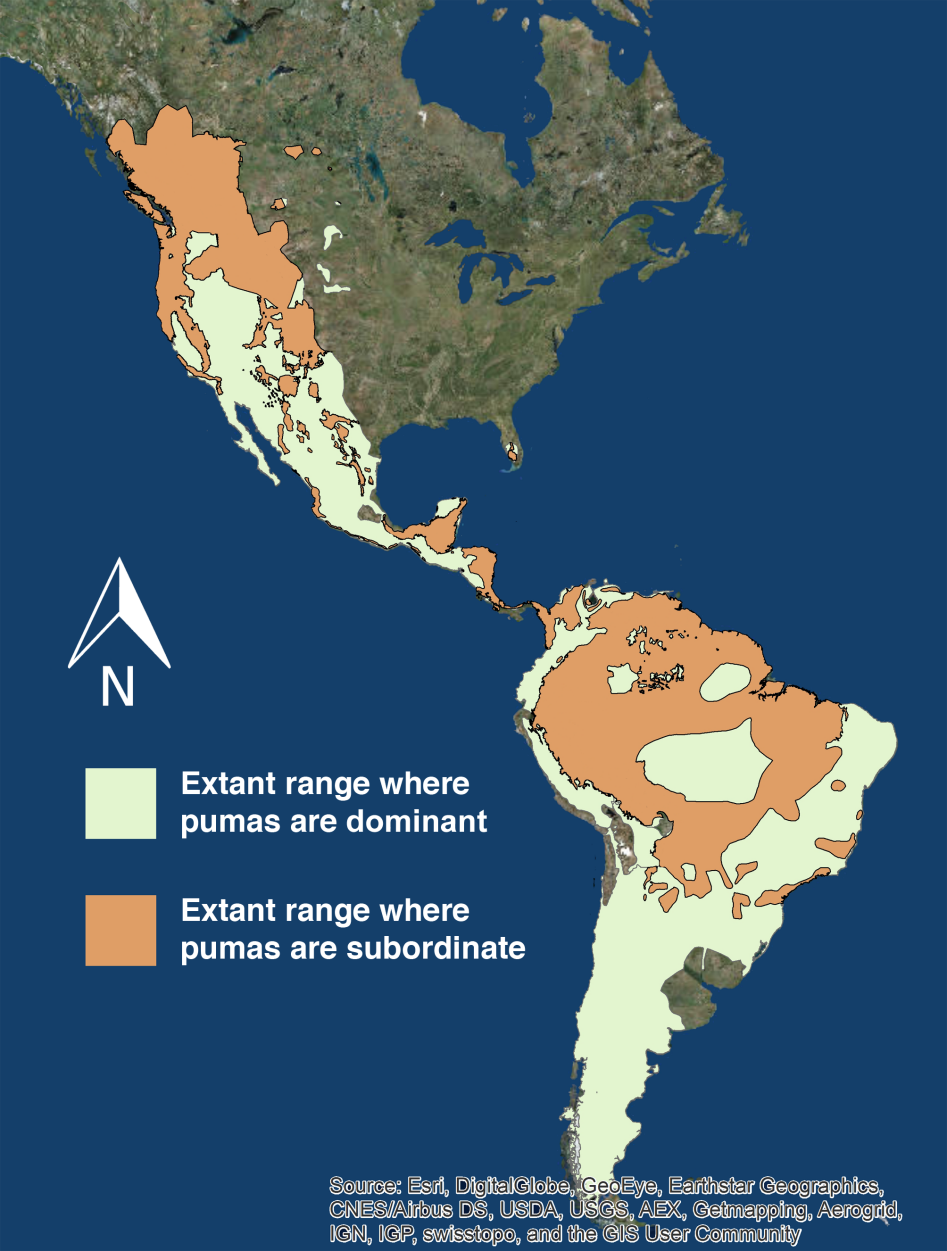
Extant puma range. The extant range of pumas in North and South America. The light green denotes the portion of puma range where they are the only or dominant apex predator, and the orange denotes the portion of puma range where they are subordinate to at minimum one other apex predator. Source: ESRI, DigitalGlobe, GeoEye, Earthstar Geographics, CNES/Airbus DS, USDA, USGS, AE, Getmapping, Aerogrid, IGN, IGP, swisstopo, CIS User Community.

Pumas are subordinate to another apex carnivore in 10,799,252 (47.5%) of their 22,735,268 km^2^ range (International Union for Conservation of Nature, 2015) across North and South America (Fig. 2).

## Discussion

Competition among carnivores in multi-species guilds is complex and difficult to disentangle (Schoener, 1983; Donadio & Buskirk, 2006; Lourenço et al., 2013). Our review provided strong evidence that pumas are both subordinate to some apex carnivores with which they are sympatric, but dominant over others. Lethal outcomes (LC) certainly provided compelling evidence of competition and subordination, but we did not rank them above NLC as non-lethal indirect effects can equally impact fitness (Ritchie & Johnson, 2009). Subordinate pumas switched their habitat use, suffered displacement at food sources, likely experienced increased energetic demands from harassment, exhibited increased starvation, and were sometimes directly killed in competitive interactions with dominant competitors. In short, pumas suffered costs when sympatric with carnivores to which they are subordinate. Thus, we suggest managers and researchers consider the potentially-additive or synergistic effects of competition and lethal management on puma population dynamics.

We found strong evidence that gray wolves are dominant over pumas, likely due to their social structure and the fact that they often have the numerical advantage over pumas (Ruth & Murphy, 2010). The effects of wolves on pumas appear numerous. Pumas exhibit habitat (Riley, Nesslage & Maurer, 2004) and dietary shifts (Elbroch et al., 2015) in the presence of wolves, and experience increased starvation as wolves re-establish in systems in which they were absent for some time (e.g., Kortello, Hurd & Murray, 2007; Elbroch et al., 2015). Wolves also harass pumas, the energy expense of which is approximately five times greater than the energy expenditure of normal hunting (Bryce, Wilmers & Williams, 2017), and kleptoparatize and displace pumas from their kills (Bartnick et al., 2013; Elbroch et al., 2017c). Wolves also directly kill all age classes of pumas (e.g., Boyd & Neal, 1992; Ruth, 2004a; Elbroch et al., 2015), even while pumas too occasionally kill wolves (e.g., Jimenez et al., 2008). Yet even with so much suggestive evidence amassing from multiple studies, we still lack for research that clearly links competition with wolves to puma fitness (e.g., survivorship, fecundity).

The influence of grizzly and black bears on pumas is less studied, but appears to be less severe than that of wolves due to the seasonal dormancy of bears that provide pumas a temporal reprieve from competition with these species. The most significant competition between bears and pumas documented in the literature is that bears displace pumas from their kills, costing pumas energetic calories in food lost and the additional efforts of hunting to procure more prey. If bears displace pumas from their kills often enough, pumas may increase their kill rates and influence other trophic levels (Elbroch et al., 2014). Research directly linking bear kleptoparatism to puma fitness, however, is lacking. For example, kleptoparatism of leopard (*Panthera pardus)* kills negatively impacts leopard reproductive success (e.g., Balme et al., 2017). At this time, we cannot currently predict whether there exists an energetic threshold at which point bear kleptoparatism impacts puma fitness, or say definitively that it does. Bears also occasionally kill puma kittens (Ruth, 2004b; LM Elbroch & HB Quigley, 2018, unpublished data), and rarely adult pumas (M Jorgensen, pers. comm., 2007). Pumas also occasionally kill black bears (e.g., Elbroch et al., 2014; Nichols, 2017; LM Elbroch & HB Quigley, 2018, unpublished data).

Jaguar-puma interactions were the most frequently studied competitive interaction among apex predators in the Americas. In contrast to wolf-puma research which has been conducted with marked individuals, telemetry, and GPS technology, most jaguar-puma research has been conducted with non-invasive camera trapping assessing spatial and temporal time-sharing of habitats, and to a lesser extent, dietary overlap through scat analysis. Such studies offer insights into competitive interactions and mechanisms of coexistence, however, this difference in methods likely accounts for the large number of publications in which researchers report that they did not detect any competition between jaguars and pumas (Table 2). Whereas some authors were certain they found evidence that jaguars were dominant over pumas (e.g., Harmsen et al., 2009), other researchers, from areas where jaguars are smaller and sometimes numerically fewer than pumas, suggested the two species may be at least equal in competitive interactions, or even that pumas may be dominant over jaguars (e.g., Gutiérrez-González & López-González, 2017). We did discover five references that report incidents of jaguars killing pumas (e.g., Crawsha Jr & Quigley, 1984; Harmsen et al., 2009), whereas we did not find a single documentation of the reverse.

Most research we uncovered suggested that pumas were dominant over coyotes, maned wolves, and felid mesocarnivores. This is not unexpected, given that pumas outweigh these species by a large margin (Donadio & Buskirk, 2006; De Oliveira & Pereira, 2014). Packs of coyotes, however, do occasionally push pumas from their kills (Harrison, 1990), as well as harass and kill puma kittens (Logan & Sweanor, 2001; Stanford, 2013). Nevertheless, there is very little research on the outcomes of competition between pumas and smaller carnivores.

Humans, of course, are also dominant over pumas. Human hunting negatively impacts puma population dynamics, as well as puma dispersal and metapopulation dynamics (Cooley et al., 2009; Stoner et al., 2013). Humans are generally the leading cause of death for pumas in non-hunted populations as well (Thompson, Jenks & , 2014; Vickers et al., 2015). At finer scales, pumas shift their activity patterns and habitat use to avoid humans and human infrastructure (Sweanor et al., 2008; Wilmers et al., 2013), as well as abandon food sources when humans are detected (Smith, Wang & Wilmers, 2015).

In the absence of human hunting, pumas self-regulate (Seidensticker et al., 1973; Wallach et al., 2015), and exhibit the complex social structures (Wallach et al., 2015; Elbroch et al., 2017b) and hunting strategies (Carbone, Teacher & Rowcliffe, 2007) that identify them as apex predators. We are, however, clearly lacking research to help us understand how pumas cope with the stressors of competition with dominant, apex carnivores. Further, we lack research that assesses the influence of human non-lethal and lethal effects on pumas simultaneous with the negative effects of competition with other sympatric carnivores. Apex predators did not evolve like prey species to contend with top-down selection pressures (Darimont et al., 2015), and therefore human-caused mortalities may be additive to other natural causes. Hunting may also influence the outcomes of competition scenarios among sympatric carnivores through the removal of key individuals (*sensu* Bischof et al., 2017). Perhaps pumas are better adapted to handle human hunting because they are already subordinate to other species. It is also possible, however, that the combination of competition with dominant carnivores combined with human harvest will lead to rapid puma declines in some populations.

## Conclusions

Managing species in complex systems is difficult, especially with cryptic species like pumas that are laborious and expensive to monitor in order to track population dynamics. Elucidating the direct and indirect effects of competition in species interactions in natural, multi-species systems is equally challenging, and a topic very much pursued in current ecology (e.g., Dröge et al., 2017; Karanth et al., 2017). Our review, if nothing else, highlights how little we know about the competitive interactions between pumas and other apex carnivores, or more generally, the costs accrued by apex predators during competitive interactions with dominant species. Therefore we encourage further research into competition among apex predators, as well as research assessing the effects of competition in managed systems where pumas, and perhaps other predators, are hunted as well. Until the time that we understand whether competitive effects are additive with human management, or even potentially synergistic resulting in unexpected, disproportionate declines in subordinate predators, we encourage caution among managers responsible for determining harvest limits for pumas and other subordinate, apex carnivores in areas where they are sympatric with dominant species. Our review, for example, suggested that pumas are negatively impacted by wolves more than grizzly bears. This is important information for managers working in regions where wolves and brown bears are recolonizing and recovering, and historic competition scenarios among multiple apex predators are being realized.

## Additional Information and Declarations

### Data Availability

The following information was supplied regarding data availability: This is a review article, and the resources exist in the public domain.
